# Acting Proactively to Manage Job Insecurity: How Worrying About the Future of One’s Job May Obstruct Future-Focused Thinking and Behavior

**DOI:** 10.3389/fpsyg.2021.727363

**Published:** 2021-10-12

**Authors:** Jessie Koen, Maarten J. van Bezouw

**Affiliations:** Department of Work and Organizational Psychology, University of Amsterdam, Amsterdam, Netherlands

**Keywords:** proactive coping, resource scarcity theory, conservation of resources theory, future focus, income, cognitive functioning, proactive career behavior, job insecurity

## Abstract

An increasing number of people experience insecurity about the future of their job, making it more important than ever to manage this insecurity. While previous research suggests that proactive coping is a promising way to alleviate job insecurity, we suggest that, paradoxically, it may be particularly difficult to act proactively when feeling emotionally distressed about the future of one’s job. Drawing on the principle of resource scarcity and the Conservation of Resources theory, we propose that affective job insecurity ignites a scarcity mindset that inhibits workers’ future focus and cognitive functioning, thereby undermining proactive career behavior. Additionally, we examine whether income adequacy can compensate for these negative consequences of job insecurity. Results of a three-wave survey study among 108 self-employed professionals during the COVID-19 pandemic showed that initial affective job insecurity was negatively related to cognitive functioning but unrelated to future focus. Yet, the latter relationship was moderated by income adequacy: affective job insecurity was positively related to future focus when participants reported high income adequacy. In turn, future focus was positively related to proactive career behavior, which was subsequently related to lower cognitive job insecurity. Thus, while replicating the finding that workers can proactively manage their cognitive job insecurity, we also showed that initial affective job insecurity may obstruct people’s cognitive functioning. We discuss how our results signal a Matthew effect, in which job insecure people with sufficient means are able to look ahead and proactively build resources to change their career, while job insecure people with insufficient means may fall behind.

## Introduction

The current world of work is characterized by great uncertainty about the future: developments such as technological change, globalization, digitalization, and increased temporary employment have contributed to increased job insecurity and decreased well-being among workers (Shoss, 2017; Jiang and Lavaysse, 2018; Lee et al., 2018). Aggravating this already uncertain world of work, the recent COVID-19 pandemic has led to a steep increase in jobs at risk, even putting a stop to some lines of work altogether. As such, being able to manage uncertainty about the continuity and stability of one’s employment (i.e., job insecurity, Shoss, 2017) has become a major theme for workers across the globe. But how does one manage such job insecurity? Despite the progress that has been made in research on job insecurity and its negative consequences, only a handful of studies have specifically focused on factors directly reducing or preventing job insecurity itself. Notwithstanding, these studies have provided the valuable insight that job insecurity can indeed be managed: individual resources and behavior, as well as organizational resources and interventions, can influence the extent to which people experience job insecurity in a given work situation (e.g., Abildgaard et al., 2018; Jiang et al., 2020b; Koen and Parker, 2020).

Promising in this regard is the notion of proactive coping with job insecurity (Klehe et al., 2012; Stiglbauer and Batinic, 2015; Probst et al., 2019; Koen and Parker, 2020). Proactive coping refers to the behaviors undertaken in advance of a potentially stressful event (e.g., job insecurity or job loss) to prevent it or to modify its form before it occurs (cf. Aspinwall and Taylor, 1997). Recent research (Koen and Parker, 2020) has shown that workers are able to decrease the feelings of job insecurity that generally arise from insecure work situations by proactively building resources to master and change one’s career (i.e., proactive career behavior). However, due to its anticipatory, self-initiated and self-directed nature, behaving proactively requires a great deal of resources, with the exertion of considerable energy, time, and attention necessary for planning and enacting (Bindl et al., 2012; Cangiano et al., 2021). This poses a problem, because job insecurity may in itself inhibit the resources that are needed to engage in proactive career behavior. Put differently, the uncertain world of work that calls for proactivity may paradoxically also obstruct people’s ability to behave proactively.

We draw on the principle of resource scarcity (Shah et al., 2012) and the Conservation of Resources theory (Hobfoll, 1989, 2001) to examine how job insecurity may affect workers’ ability to engage in proactive career behavior. Central to both the principle of resource scarcity and the Conservation of Resources theory is that people have a limited amount of resources (e.g., time, money, energy). Once these resources become threatened or depleted, people start to focus on short-term solutions to protect and/or regain current resources, rather than on long-term solutions to create new and/or alternative resources. Given that experiencing job insecurity threatens and depletes one’s resources (De Cuyper et al., 2012; Richter et al., 2020), we argue that job insecure workers are unlikely to engage in long-term oriented proactive career behaviors that may help to create a more secure future.

**FIGURE 1:**
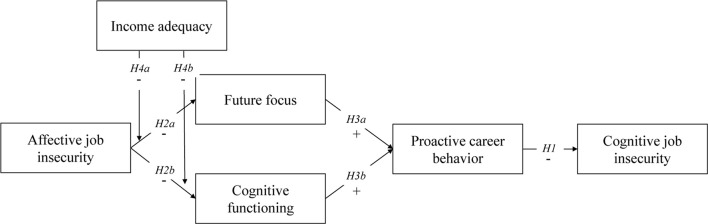
Hypothesized model.

Specifically, we argue that initial worries and emotional distress about potential job loss (i.e., affective job insecurity, Greenhalgh and Rosenblatt, 1984; Hellgren et al., 1999; Huang et al., 2012) ignites a scarcity mindset that (a) negatively affects workers’ ability to focus on the future and (b) inhibits workers’ cognitive functioning. Moreover, we argue that future focus and cognitive functioning are the very things that are necessary to engage in proactive career behavior and, hence, to decrease the expected likelihood of losing one’s job (i.e., cognitive job insecurity, cf. Huang et al., 2012; Vander Elst et al., 2014). Importantly, for potential interventions, we also examine whether income adequacy (i.e., having sufficient income to make ends meet) can help to mitigate the negative effects of affective job insecurity on future focus and cognitive functioning, and, consequently, on proactive career behavior and subsequent cognitive job insecurity. Figure 1 presents our research model.

### Proactive Coping With Job Insecurity

Insecurity about the future of one’s job is one of the most common stressors in the work place (De Witte et al., 2015; Shoss, 2017; Lee et al., 2018). Research has shown that experiencing job insecurity has negative consequences for people’s mental and physical health, for their job performance, and even for their career prospects (Sverke et al., 2002; Cheng and Chan, 2008; De Witte et al., 2016; Jiang and Lavaysse, 2018). It is therefore not surprising that job insecurity research has focused on uncovering ways in which workers can minimize the stress and strain that typically results from job insecurity. This “traditional” or reactive perspective on coping, however, views job insecurity as an existing threat and workers as passive respondents to their environment who can only influence the consequences of that threat. Yet, workers can also be considered as active agents who can influence their own job security by improving current employment circumstances or creating new career opportunities (Crant, 2000; Strauss et al., 2012; Koen and Parker, 2020). In this alternative proactive perspective on coping, workers are able to decrease, minimize, or even prevent the likelihood of job loss by approaching it proactively.

While “traditional” or reactive coping is aimed at minimizing negative consequences of an existing threat, proactive coping aims to reduce the threat itself (Aspinwall and Taylor, 1997; Stiglbauer and Batinic, 2015). More specifically, proactive coping refers to future-oriented coping that tries to detect and proactively manage stressors before they can fully develop (Aspinwall and Taylor, 1997). It involves building resources and acquiring skills that are not necessarily needed to address a current threat but, rather, to prepare for the longer term when potential threats may occur. By coping proactively –through emotions, thoughts, and behaviors– people can tackle the threat in its early stages rather than cope with the consequences of the threat in its full-blow state (Aspinwall, 2011). An example of proactive coping can be to develop technical skills that may be needed in a future job, or to build a network that can help to signal new job- and career opportunities.

The proactive perspective on job insecurity has received increased attention in the past few years. At its core, this research assumes that people are able to prevent or lessen the likelihood job loss, and thus the experience of job insecurity, itself. For example, Abildgaard et al. (2018) examined the effect of an intervention aimed to alleviate employees’ experiences of job insecurity during organizational restructuring. They found a slower increase in job insecurity among employees who participated in the intervention compared to those who did not participate in the intervention, and argued that this was because the intervention fostered a proactive stance toward restructuring (also see Sverke et al., 2008). Another example comes from a study by Probst et al. (2019). They examined whether job insecurity prompted impression management behaviors or vice versa, and found the latter: employees who proactively engaged in impression management techniques at work experienced lower subsequent levels of cognitive job insecurity. Likewise, in a sample of workers whose temporary contract was close to expiring, Koen and Parker (2020) found that temporary workers who engaged in proactive career behavior experienced lower levels of cognitive job insecurity than their less proactive counterparts. Taken together, these studies suggest that insecure work situations such as organizational restructuring and temporary contracts are not necessarily threatening; rather, workers’ experience of job insecurity in such situations seem to depend on their proactive coping efforts.

Thus, research on proactive coping with job insecurity suggests that workers can proactively create opportunities to keep their job or to find a comparable job elsewhere, and, hence, manage their cognitive job insecurity (see also Stiglbauer and Batinic, 2015). As such, we expect that engaging in proactive career behavior should help workers to decrease the perceived risk of job loss –their cognitive job insecurity.

*H1: Proactive career behavior will be negatively related to subsequent cognitive job insecurity.*

At the same time, as we will argue next, we expect that the initial experience of being worried or emotionally distressed about potential job loss (i.e., affective job insecurity, Greenhalgh and Rosenblatt, 1984; Hellgren et al., 1999; Huang et al., 2012) may inhibit people’s proactive career behavior, and therefore their ability to manage their subsequent job insecurity.

### How Initial Job Insecurity May Inhibit Proactive Career Behavior

We expect that there is a paradox to proactive coping with job insecurity. While proactive career behavior may help workers to alleviate the experience of (cognitive) job insecurity, it may be particularly difficult to act proactively when feeling emotionally distressed about the future of one’s job, i.e., when experiencing affective job insecurity. That is, proactivity is an effortful and future-focused behavior that requires sufficient energy and resources (Grant and Ashford, 2008; Cangiano et al., 2021). To be able to engage in such proactive behavior, people need resources to think and plan ahead, beyond what is needed for more urgent tasks and events (Halbesleben and Bowler, 2007; Parker et al., 2013; Schmitt et al., 2017). Yet, as we will argue next, these resources tend to be threatened when experiencing emotional distress and fear about the future of one’s job: such job insecure workers are more likely to invest their time and energy in dealing with potential job loss directly than to engage in proactive behaviors that can help to master and change their career and job security in the long term. As such, we expect that affective job insecurity will inhibit people’s proactive career behavior.

To explain how affective job insecurity may inhibit proactive career behavior, we combine research in behavioral economics (i.e., resource scarcity, Shah et al., 2012) with research in organizational psychology (i.e., the Conservation of Resources theory, Hobfoll, 1989, 2001). Although developed in separate disciplines, both lines of research center around the idea that a lack of resources can impair cognitive functioning, and that such a lack is conducive to a tendency to focus on short-term rather than long-term solutions. We apply this same principle here: we view initial feelings of affective job insecurity as a signal that resources are threatened or already depleted, which may impair people’s ability to focus on the future as well as their cognitive functioning.

According to the principle of resource scarcity, a current lack of resources changes how people approach problems and make decisions (Shah et al., 2012). Scarcity of any kind of resources (money, time, food) directs people’s attention to the current threat and away from other, more long-term, threats and problems. For example, when money is scarce, people tend to focus on buying weekly groceries rather than on paying next month’s rent. Likewise, when time is scarce, people tend to focus on meeting tomorrow’s deadline rather than on preparing for an assignment that is due next week (Shah et al., 2012). In addition to the attentional shift from a long-term focus to a short-term focus, the preoccupation with a pressing problem can consume people’s cognitive resources, leaving less room for other tasks (Mani et al., 2013). Put differently, resources such as attention and energy are finite and once they are used for one domain they become unavailable for other domains (see also resource drain theory, Edwards and Rothbard, 2000). For example, an air traffic controller who is focused on preventing a potential collision course loses cognitive capacity to coordinate other planes in the air (Mani et al., 2013). Here, we propose that affective job insecurity induces a situation of resource scarcity that will direct people’s attention away from the long term and will deplete their cognitive functioning, thereby undermining their ability to engage in future-oriented activities such as proactive career behavior.

The assumption that affective job insecurity induces a situation of resource scarcity that inhibits proactive career behavior is in line with the Conservation of Resources theory (Hobfoll, 1989, 2001), a theory that is often used in the job insecurity literature to explain how emotional distress about the future of one’s job can result in negative consequences such as exhaustion and burnout symptoms (e.g., De Cuyper et al., 2012). The Conservation of Resources theory explains human behavior based on the need to conserve resources. The central tenet of this theory is that the maintenance or increase of resources is associated with well-being, while a threat of resource loss or an actual decline in resources is related to stress and strain. Additionally, a threat to resource loss evokes a focus upon short-term resource conservation rather than on long-term resource creation (Hobfoll, 1989, 2001). When resources are threatened, people seek to protect their resources by putting less effort into behaviors they are not required to perform or that may not pay off in the short term (Halbesleben and Bowler, 2007). Similar to the principle of resource scarcity (Shah et al., 2012), such resource protection may thus trigger an increased focus on the core task (Halbesleben and Bowler, 2007).

Given that proactive behavior requires energy and cognitive resources beyond the resources that are required for core tasks (Bolino et al., 2010; Parker et al., 2013; Schmitt et al., 2017), it is unlikely that workers will engage in proactive career behavior when worrying about the future of their job. That is, when people experience job insecurity, one of their most essential resources (i.e., employment) is threatened (De Cuyper et al., 2012; Richter et al., 2020). This feeling of job insecurity, or more specifically the worrying about the future of one’s job (i.e., affective job insecurity), requires energy: people who ruminate and dwell on the possible loss of their job are severely drained of energy (Richter et al., 2020). As such, Conservation of Resources theory would predict that workers who experience affective job insecurity resort to protecting further loss of resources: they will more effectively allocate their remaining resources to ensure optimal functioning and, hence, are less likely to invest their energy into resource-consuming behaviors such as proactivity (Halbesleben and Bowler, 2007; Bolino et al., 2010; Parker et al., 2013; Schmitt et al., 2017).

Thus, based on both the principle of resource scarcity (Shah et al., 2012) and the Conservation of Resource theory (Hobfoll, 1989, 2001), we expect that initial worries and emotional distress about the future of one’s job (i.e., affective job insecurity) will inhibit the very resources that are necessary to engage in proactive career behavior: a focus on the future and cognitive functioning.

*H2: Affective job insecurity will be negatively related to (a) future focus and (b) cognitive functioning.*

In turn, we expect that an impaired future focus and impaired cognitive functioning will inhibit proactive career behavior. This expectation is based on the definition of proactivity: proactive behavior is self-directed and future-focused behavior in which an individual aims to bring about change (Bindl and Parker, 2010; Parker et al., 2010). This definition bears two important elements for our expectations. First, due to its anticipatory and self-directed nature, behaving proactively requires a great deal energy, time and attention for planning and enacting (Grant and Ashford, 2008; Bindl et al., 2012). As such, we expect that higher levels of cognitive functioning will be associated with higher levels of proactive career behavior. Second, proactivity is future-focused: it involves anticipating, thinking ahead and taking actions for the future (Bindl and Parker, 2010; Parker et al., 2010). Theoretically, then, future-oriented thinking should positively contribute to proactive behavior (Aspinwall, 2011; Wu et al., 2013). Indeed, Parker and Collins (2010) showed that consideration of future consequences was positively related to proactive work behaviors, and Strauss et al. (2012) showed that a focus on the self in the longer-term future (“future work self”) stimulated proactive skill development, which involves building career-relevant resources and skills for the future. As such, we expect that a stronger focus on the future –defined here as the allocation of attention to the future (Shipp et al., 2009)– will be associated with higher levels of proactive career behavior.

*H3: (a) Future focus and (b) cognitive functioning will be positively related to subsequent proactive career behavior.*

### The Moderating Role of Income Adequacy

An important assumption in the Conservation of Resources theory (Hobfoll, 1989, 2001) is that individuals are embedded within their social contexts, and that these contexts can further threaten their resources. Those who lack resources in their (social) context are more vulnerable to resource loss while those who possess or have access to resources in their (social) context are less vulnerable to resource loss. Following this reasoning, certain resources may compensate for the resource loss associated with job insecurity, thereby buffering its negative consequences. That is, the threat of losing resources associated with employment (e.g., income, social support, doing something meaningful; cf. Jahoda, 1982) may be less detrimental when one of those resources is compensated for in one’s social context. For example, the possibility of losing one’s job may be less threatening when people have sufficient financial means to pay their rent or when they have a strong social network that they can call on for support and help. The experience of job insecurity, then, may be less likely to result in stress and decreased well-being. Indeed, Lim (1996) showed that having access to a supportive system can buffer the negative effect of job insecurity on life satisfaction and Jiang and Probst (2017) showed that job insecurity was less likely to result in burnout symptoms in countries with low income inequality –a social context in which people have more access to resources such as employment protection, labor standards, unemployment benefits (Zafirovski, 2005).

Here, we propose that income adequacy (i.e., having sufficient income to make ends meet) represents a resource that can compensate for the negative consequences of affective job insecurity. This proposition has its roots in research on “flexicurity” (an European employment strategy that combines security for workers with flexibility for organizations, see Wilthagen and Tros, 2004; Muffels and Wilthagen, 2013). The flexicurity strategy suggests that income security can act as a compensating mechanism for job insecurity in a labor market characterized by uncertainty, as it ensures that financial needs are met during a period of unemployment through unemployment insurance and/or social security benefits. For example, Sjöberg (2010) showed that generosity of unemployment benefits within a country contributed positively to workers’ well-being, especially those in insecure work situations.

We argue that income adequacy will mitigate the negative effect of initial affective job insecurity on people’s future focus as well as on their cognitive functioning. Specifically, we expect that worrying about one’s job will be less detrimental to people’s cognitive functioning and their future focus when they have sufficient income to make ends meet. Although having a sufficient income may not directly take away people’s emotional distress about the future of their job, such income adequacy does provide one less worry: the worry about being able to make ends meet. In terms of Conservation of Resources theory, having a sufficient income means that there is one less resource that is threatened by job insecurity. This would suggest that job insecure people with sufficient income are less vulnerable to further resource loss and, thus, that worrying about the future of their job will be less likely to impair their future focus and cognitive functioning. Likewise, in terms of the principle of resource scarcity (Shah et al., 2012), having a sufficient income implies that people will experience less resource scarcity, which should mitigate the negative effects of job insecurity on their long-term focus and cognitive functioning. Earlier research provides some initial evidence for this assumption in that worries about having sufficient income can lead to decreased cognitive capacity (Meuris and Leanaa, 2018). We thus expect that:

*H4: The relationship between affective insecurity and (a) future focus and (b) cognitive functioning will be moderated by income adequacy, in such a way that the hypothesized negative relationships will become weaker when income adequacy is higher.*

## Materials and Methods

### Design and Context

In this study, we assumed that initial affective job insecurity would be associated with people’s future focus and cognitive functioning, which would in turn affect their proactive career behavior and subsequent cognitive job insecurity (see also Figure 1). To model these presumed sequential relationships, we conducted a three-wave survey study in which we assessed all variables in the hypothesized model at Time 1, proactive career behavior at Time 2, and cognitive job insecurity at Time 3. This allowed us to control for people’s prior levels of these outcome variables and, as such, to rule out that the results were driven or altered by people’s initial proactive career behavior and/or initial cognitive job insecurity.

In addition, we hypothesized that income adequacy would mitigate the negative effects of affective job insecurity on future focus and cognitive functioning. We therefore used a sample of participants that were likely to vary in their income adequacy, i.e., the extent to which they had sufficient income to make ends meet. Specifically, we surveyed self-employed professionals during the COVID-19 pandemic in 2020–2021 who received governmental financial support. In the country in which this study was conducted, all self-employed professionals who had gotten into financial difficulties due to the COVID-19 crisis were eligible to apply for governmental financial support. Eligibility criteria included having an income below the national social minimum (€1.503,31 per month) due to the COVID-19 crisis; being an established self-employed professional between 18 and 67 years old; owning a company registered at the Chamber of Commerce; and having worked at least 1225 h as a self-employed professional in 2019. The maximum amount of financial support that self-employed professionals could receive for a period of 6 months equaled the national social minimum referred to above. Whether this amount of financial support was sufficient to make ends meet depended on participants’ fixed monthly expenses (e.g., rent, electricity and water, office supplies and software licenses, insurances, etc.). For example, a self-employed participant with expensive office space was less likely to be able to make ends meet with the amount of financial support compared to a self-employed participant who worked from a home office. As such, participants in our sample were likely to vary in their level of income adequacy.

### Sample and Procedure

After IRB approval (2020-WOP-12462), we conducted a three-wave survey study among self-employed professionals during the COVID-19 pandemic in 2020–2021. Participants were contacted *via* the governmental agency that provided financial support and received three consecutive online questionnaires each set 2 months apart. To enhance our response rate, participants received a digital coupon of €5 for a purchase in a leading online store if they completed one questionnaire, €10 if they completed two questionnaires, and €25 if they completed all three questionnaires. Participants were included in the study when they (a) received governmental financial support for self-employed professionals, (b) were between 18 and 65 years old, and (c) worked at least 20 h per week on average before the onset of the COVID-19 pandemic.

A total of 171 participants completed the questionnaire at Time 1, of which 91 completed the questionnaire at Time 2 (53.2%) and 108 completed the questionnaire at Time 3 (63.2%). The overall sample of participants with complete data at all three time points consisted of 53.8% men and 46.2% women. Participants’ average age was 44.3 years (*SD* = 13.1), and 23.1% had completed a vocational training degree whilst 47.3% had a bachelor or master degree. The remaining 29.6% indicated that they had a high school degree or a different type of degree. Participants worked in a wide variety of industries, with the largest share of participants working in the cultural sector (21.3%), followed by 13.9% in the catering or hospitality industry, 6.5% in education, 4.6% in retail, 4.6% in financial services, and 4.6% in ICT; the remaining 44.5% worked in other industries such as transportation, cleaning, service work, or construction. On average, participants had 11.4 years of work experience as a self-employed professional (*SD* = 11.0) and worked 37.5 h per week before the onset of the COVID-19 pandemic (*SD* = 11.9), which had dropped to 17.2 h per week (*SD* = 15.8) at the time of our study.

### Measures

Unless indicated otherwise, all variables were assessed with existing and validated 7-point Likert scales, ranging from 1 [*strongly disagree*] to 7 [*strongly agree*]. Table 1 presents the internal consistency (Cronbach’s alpha) of each variable.

**TABLE 1:** Means, Standard Deviations, Internal Consistencies, and Correlations.

	M	SD	1	2	3	4	5	6	7	8	9	10	11	12
1. Age (T1)	44.11	12.93	*(–)*											
2. Gender^1^ (T1)	1.39	0.50	−0.19**	*(–)*										
3. Education^2^ (T1)	4.52	1.43	0.00	0.20**	*(–)*									
4. Income adequacy (T1)	2.17	0.98	−0.22**	0.18**	0.03	*(–)*								
5. Financial buffer^3^ (T1)	5.57	7.57	0.02	0.10	0.20**	0.18**	*(–)*							
6. Affective job insecurity (T1)	4.57	1.61	−0.23**	0.21**	–0.12	–0.15	−0.27**	*(0.85)*						
7. Cognitive functioning (T1)	5.24	0.99	0.23**	−0.33**	–0.04	–0.02	0.08	−0.47**	*(0.84)*					
8. Future focus (T1)	4.89	1.24	−0.30**	–0.09	–0.00	0.05	–0.07	0.06	0.02	*(0.91)*				
9. Proactive career behavior (T1)	3.88	1.41	−0.32**	–0.05	0.12	–0.04	0.03	–0.09	0.01	0.45**	*(0.93)*			
10. Proactive career behavior (T2)	3.80	1.44	−0.32**	0.19	0.13	–0.03	–0.11	–0.02	–0.06	0.39**	0.80**	*(0.93)*		
11. Cognitive job insecurity (T1)	4.05	1.41	−0.26**	0.09	–0.14	−0.22**	−0.21**	0.70**	−0.38**	0.09	0.00	0.10	*(0.83)*	
12. Cognitive job insecurity (T3)	3.86	1.49	–0.19	0.04	–0.11	–0.16	–0.15	0.48**	−0.34**	0.09	–0.07	–0.16	0.60**	*(0.87)*

**p < 0.05 (2-tailed).

*^1^Categories include 1 = male; 2 = female.*

*^2^Categories include 1 = none; 2 = elementary school; 3 = high school; 4 = vocational training; 5 = bachelor degree at university of applied sciences; 6 = bachelor degree at university; 7 = master degree.*

*^3^Number of months that participants expected to be able to make ends meet with their current financial buffer (e.g., savings, real estate).*

*The sample at T1 included *N* = 171 participants, the sample at T2 included *N* = 91 participants, the sample at T3 included *N* = 108 participants. Internal consistencies are presented at the diagonal.*

#### Affective Job Insecurity

Participants’ affective job insecurity was assessed at Time 1 with Hellgren et al.’s (1999) 3-item scale. An example item is: *“I am worried about having to leave my job before I would like to.”*

#### Income Adequacy

At Time 1, we assessed income adequacy with one item, by asking participants to indicate on a 5-point Likert scale to what extent their current income (including the governmental financial support that they received) was sufficient to make ends meet every month (ranging from 1 [*completely insufficient*] to 5 [*more than sufficient*]).

#### Future Focus

Future focus was assessed at Time 1 with Shipp et al.’s (2009) temporal focus scale. We used the 4 items that referred to future focus, e.g., *“I think about what my future has in store.”* Participants were asked to indicate on a 7-point frequency scale to what extent they had thought about the future as indicated by the item (1 [*never*]; 3 [*sometimes*]; 5 [*frequently*]; 7 [*constantly*]).

#### Cognitive Functioning

Participants’ cognitive functioning was assessed at Time 1 with eight items from the CAT-PD project that referred to cognitive problems (cf. Simms et al., 2011). Participants were asked to indicate on a 7-point frequency scale to what extent they had experienced difficulties with cognitive functioning in the past month (1 [*never*]; 3 [*sometimes*]; 5 [*frequently*]; 7 [*constantly*]). Items were coded in reverse to reflect cognitive functioning rather than cognitive problems.

#### Proactive Career Behavior

Proactive career behavior was assessed at Time 1 and Time 2 with a scale previously used by Strauss et al. (2012) and Koen and Parker (2020). This scale originally contains four subdimensions of proactive career behavior: career planning (4 items, e.g., *“I am planning what I want to do in the next few years of my career”*), career consultation (3 items, e.g., *“I initiate talks with my supervisor about training or work assignments I need to develop skills that will help my future work chances”*), skill development (3 items, e.g., *“I develop skills which may not be needed so much now, but in future positions”*) and networking (3 items, e.g., *“I am building a network of contacts or friendships to provide me with help or advice that will further my work chances”*). Because participants in the current study were self-employed, we omitted the 3 items referring to consulting one’s supervisor or manager (i.e., career consultation) from the scale to form the variable proactive career behavior.

#### Cognitive Job Insecurity

We assessed participants’ cognitive job insecurity at Time 1 and Time 3 with Vander Elst et al.’s (2014) 4-item scale. Example items are *“I think I might lose my job in the near future”* and *“I am sure I can keep my job”* (reverse coded).

#### Demographic and Control Variables

Meta-analytical evidence suggests that the demographic variables age, gender and level of education are correlated with perceived job insecurity (cf. Sverke et al., 2002; Cheng and Chan, 2008). We therefore included these variables as demographic control variables in the current study. Level of education was operationalized as participants’ highest completed degree, ranging from 1 [*none*], 2 [*elementary school*], 3 [*high school*], 4 [*vocational training*], 5 [*bachelor degree at a university of applied sciences*], 6 [*bachelor degree at a university*] to 7 [*master degree*]. In addition, we assessed the number of months that participants expected to be able to make ends meet with their current financial buffer (e.g., savings, real estate) and included this financial buffer as a control variable.

## Results

Table 1 presents the means, standard deviations, and correlations between all variables in this study.

### Measurement Model

We conducted a Confirmatory Factor Analysis (CFA) in AMOS 25.0 to evaluate the construct validity of the scales. For the independent variables assessed at Time 1, we compared the hypothesized five-factor model (i.e., a model in which the items of affective job insecurity, cognitive job insecurity, proactive career behavior, future focus, and cognitive functioning loaded on their respective latent factor) to a four-factor model (i.e., a model in which the items of affective job insecurity and cognitive job insecurity loaded on one latent factor while proactive career behavior, future focus and cognitive functioning loaded on their respective latent factor) and to a common-factor model (i.e., a model in which all items loaded on one latent factor). The error terms of proactive career behavior were allowed to covary within their respective dimension. Results showed that the five-factor model yielded an acceptable fit, χ2/*df* = 1.86, *p* = 0.00, NFI = 0.79; CFI = 0.89, RMSEA = 0.07, and fitted the data significantly better than the four-factor model, Δχ2(4) = 22.32, *p* = 0.00, or the common-factor model, Δχ2(10) = 1225.79, *p* = 0.00.

For the outcome variables, we compared the hypothesized two-factor model (i.e., a model in which the items of proactive career behavior at Time 2 and cognitive job insecurity at Time 3 loaded on their respective latent factor) to a common-factor model (i.e., a model in which all items loaded on one latent factor). Results showed that the two-factor model yielded a good fit, χ2/*df* = 1.70, *p* = 0.00, NFI = 0.90; CFI = 0.96, RMSEA = 0.08, and fitted the data significantly better than the common-factor model, Δχ2(1) = 85.14, *p* = 0.00.

### Hypotheses Testing

**FIGURE 2:**
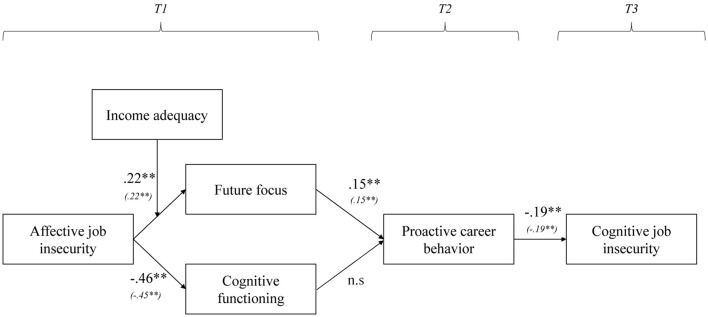
Results of path modeling. Proactive career behavior (T2) and cognitive job insecurity (T3) were controlled for their associated level at T1. The hypothesized model was also tested with the demographic control variables age, gender, level of education, and financial buffer as covariates of all Time 1 variables. The results of the model with demographic control variables (χ2/*df* = 2.16, *p* = 0.00, CFI = 0.89, RMSEA = 0.06) are displayed between brackets.

We examined the hypothesized model (see Figure 1) using path modeling in SPSS AMOS 25.0. For each outcome variable (i.e., proactive career behavior at Time 2 and cognitive job insecurity at Time 3), we controlled for participants’ prior level of the respective variable at Time 1. To optimize statistical power of the model, we used an estimate means procedure for participants who responded to Time 1 and Time 3 but had missing values at Time 2. The hypothesized model was tested with and without demographic control variables. In the model with control variables, we included age, gender, level of education and financial buffer as correlates of all Time 1 variables. The results of the hypothesized model without demographic control variables were the same as the results of the hypothesized model without demographic control variables (see also Figure 2). In line with Becker’s (2005) recommendations, we therefore omitted the demographic control variables from our analyses to avoid any unnecessary decline in statistical power. Thus, the results reported below are the results from the hypothesized model without demographic control variables.

Results of the path analyses are presented in Figure 2. The hypothesized model showed a good fit to the data, χ2/*df* = 2.49, *p* = 0.00, CFI = 0.92, RMSEA = 0.06.

Hypothesis 1 stated that proactive career behavior would negatively affect participants’ later cognitive job insecurity. Results confirmed this Hypothesis and showed that proactive career behavior at T2 was negatively related to cognitive job insecurity at T3, after controlling for cognitive job insecurity at T1 (Est_std_ = −0.19, *p* = 0.02).

Hypothesis 2 stated that affective job insecurity would negatively affect participants’ (a) future focus and (b) cognitive functioning. Results showed no support for Hypothesis 2a: affective job insecurity was unrelated to future focus. Results did support Hypothesis 2b: affective job insecurity was negatively related to cognitive functioning (Est_std_ = −0.46, *p* < 0.01).

Hypothesis 3 stated that (a) future focus and (b) cognitive functioning would be positively associated with participants’ proactive career behavior. Results supported Hypothesis 3a: future focus was positively related to proactive career behavior at T2, after controlling for proactive career behavior at T1 (Est_std_ = 0.15, *p* = 0.03). Yet, cognitive functioning was unrelated to proactive career behavior at T2 (after controlling for proactive career behavior at T1), providing no support for Hypothesis 3b.

**FIGURE 3:**
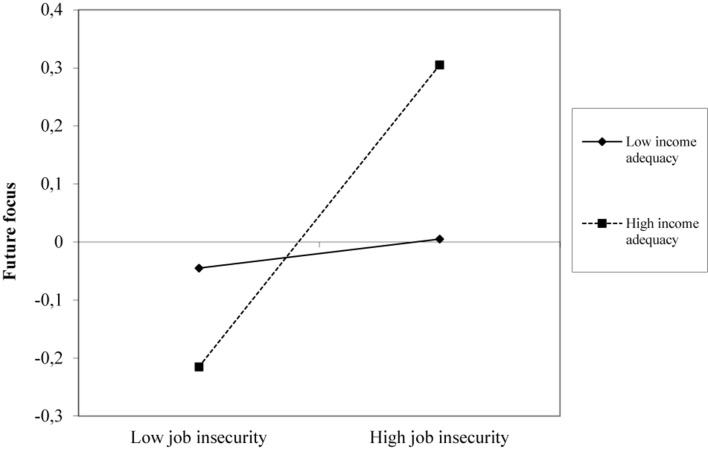
Interaction effect between affective job insecurity and income adequacy on participants’ future focus.

Hypothesis 4 stated that income adequacy would mitigate the presumed negative relationships between affective job insecurity and (a) future focus and (b) cognitive functioning. Results showed that income adequacy did indeed moderate the relationship between affective job insecurity and future focus (Est_std_ = 0.22, *p* = 0.03), but not in the expected direction. As shown in Figure 3, higher affective job insecurity was positively rather than negatively related to a stronger future focus, but only when participants reported that it was easy to make ends meet with their current income (i.e., high income adequacy). Additionally, results showed no interaction effect of affective job insecurity and income adequacy on cognitive functioning. Thus, these results do not support Hypothesis 4a, nor Hypothesis 4b.

## Discussion

The rapidly changing labor market and recent COVID-19 pandemic have led to a steep increase in job insecurity across the world. Given the negative consequences of job insecurity for people’s health, well-being, and careers (cf. Shoss, 2017; Lee et al., 2018), it is vital that workers are able to manage such feelings of job insecurity. The results of this three-wave survey study show that managing job insecurity in times of great uncertainty is easier said than done. Although we replicated the finding that workers can proactively minimize their own cognitive job insecurity (cf. Koen and Parker, 2020), and uncovered that future focus is an important determinant of such proactive career behavior, we also showed that initial affective job insecurity inhibited people’s cognitive functioning: the more people worried about the future of their job, the more likely they were to experience difficulties with thinking straight and formulating ideas clearly. Yet, affective job insecurity did not restrict people’s future focus; rather, it prompted future focus among those who did not have to worry about their income. Taken together, these findings suggest that worrying about potential job loss may impair people’s cognitive functioning but may not necessarily undermine their ability to engage in future-oriented activities such as proactive career behavior. In fact, worrying about potential job loss may even stimulate proactive career behavior when people’s income is secure.

### Theoretical Implications and Directions for Future Research

This study makes five important contributions to the literature. First, by adopting a proactive perspective to coping with job insecurity, we were able to show that workers can manage their feelings of job insecurity. Specifically, after controlling for initial cognitive job insecurity, greater engagement in proactive career behavior was associated with lower cognitive job insecurity a few months later. Thus, when workers are proactively creating career opportunities through, for example, investing in their network or developing skills that they may need in future jobs, they can decrease the expectation that job loss will happen in the near future. This finding adds to a growing body of literature on proactive coping with job insecurity: our results substantiate the idea that workers can –rather than just decreasing the negative consequences of job insecurity– also tackle the problem at its roots and prevent or lessen the experience of job insecurity itself (Abildgaard et al., 2018; Probst et al., 2019; Jiang et al., 2020b; Koen and Parker, 2020). We believe that this is a particularly relevant avenue for future research, given that job insecurity is becoming more of a rule than an exception in the current world of work. Hence, we encourage researchers to further explore ways in which workers may proactively prevent cognitive job insecurity, and, as such, its negative consequences.

Second, this study adds to the job insecurity literature that distinguishes between affective job insecurity (i.e., emotional distress about losing one’s job) and cognitive job insecurity (i.e., the expected likelihood of losing one’s job). Despite a generally positive relation between both types of insecurity (e.g., Huang et al., 2012), this line of research has demonstrated that cognitive and affective job insecurity are conceptually and empirically distinct constructs (e.g., Jiang and Lavaysse, 2018). In fact, recent findings suggest that the positive relation between cognitive job insecurity on the one hand and affective job insecurity on the other hand can be conditional on other factors: two people who have similar expectations about the likelihood of job loss do not necessarily experience the same levels of emotional distress about that job loss (Jiang et al., 2020a). Yet, in this view, affective job insecurity is often considered a consequence of cognitive job insecurity (Huang et al., 2012); the expectation that it is likely to lose one’s job triggers an emotional reaction. The current findings corroborate the nuance in the relation between cognitive and affective job insecurity by showing that proactive career behavior can indirectly reduce cognitive job insecurity a few months after experiencing affective job insecurity, yet, the findings also open up our thinking about the directionality of the relationship between both types of insecurity. That is, we find that affective job insecurity indirectly affects proactive career behavior aimed at reducing cognitive job insecurity, signaling the need examine potential recursive effects between affective and cognitive job insecurity as well as potential mediators and moderators within these dynamics. Put differently, the emotional reaction to potential job loss may indirectly influence the likelihood of job loss through people’s efforts to change the insecure situation. We believe that a longitudinal (diary) design may be able to capture these dynamic and recursive processes between cognitive job insecurity and affective job insecurity, and, perhaps, their impact on (career) behaviors, well-being, and actual job loss.

Third, our findings extend research on proactive coping with job insecurity by uncovering determinants of proactive career behavior. We found that future focus was an important determinant of proactive career behavior: people who were able to allocate their attention to the future were more likely to engage in proactive career behavior. This finding is in line with previous findings that show that focusing on a long-term future stimulates proactivity aimed at accumulating future resources (Parker and Collins, 2010; Strauss et al., 2012; Strauss and Parker, 2015). Yet, opposite to what is often assumed in proactivity research, such future focus is not a stable individual trait but rather depends on the situation: our results showed that feelings of job insecurity could actually prompt a future focus when people had an adequate income. This finding may be explained by the transactional theory of stress and coping (Lazarus and Folkman, 1984), which posits that a situation can be appraised as a loss, challenge, or threat, depending on a combination of person and situation factors. In the case of adequate income –or the security of sufficient financial support to cover one’s fixed expenses for a few months–, feelings of job insecurity might be appraised as a challenge rather than as a threat, and, as such, may induce a future focus and subsequent proactive career behavior. Additionally, we found that cognitive functioning was not an antecedent of proactive career behavior. This finding is surprising, given the idea that behaving proactively requires a great deal cognitive resources such as energy, time and attention (Grant and Ashford, 2008; Bindl et al., 2012). Our measure of cognitive functioning may not have reflected such cognitive resources properly. Alternatively, impaired cognitive functioning may generate different behaviors: behaviors aimed to protect against further cognitive resource loss rather than behaviors aimed to protect against loss of employment. Indeed, workers threatened with loss of resources tend to be focused on acquiring new resources but will not invest in just any type of resources –only those resources that can help them to replenish their threatened resources (Halbesleben and Bowler, 2007; Breevaart and Tims, 2019). While proactive career behavior can, as evidenced by our results, help to protect against the threat of job loss by creating more job security, it may not help to overcome the threat of cognitive resource loss and may thus not have been affected by impaired cognitive functioning.

Fourth, we introduced the idea that there may be a paradox to proactive coping with job insecurity. Drawing on the principle of resource scarcity (Shah et al., 2012) and the Conservation of Resources theory (Hobfoll, 1989, 2001), we expected a “loss spiral” (Hobfoll, 1989, 2001) where initial emotional distress about the future of one’s job would obstruct the very resources needed to engage in proactive career behavior, thereby inhibiting the possibility to proactively manage one’s future job insecurity. Although we showed that initial affective job insecurity inhibited cognitive functioning, and that a future focus was necessary to engage in proactive career behavior which in turn decreased subsequent feelings of job insecurity, we did not find evidence for the full loss spiral implied in our study. A possible explanation for the lack of this loss spiral is that job insecurity was closely related to the COVID-19 pandemic in our study. For participants, job insecurity may therefore have felt as a rather temporary threat that could be overcome by short-term solutions. Uncovering the full extent to which loss and gain spirals apply to job insecurity requires additional research in different contexts, as well as the employment of different methods. We believe that a longitudinal diary design or cross-lagged panel design that examines the dynamic within-person process between job insecurity and proactivity can move the field forward.

Fifth, we found that job insecure people with a sufficient income were more likely –instead of less likely– to focus on a long-term future, and therefore better able to proactively manage their feelings of job insecurity. Put differently, for those who experienced income security, initial worries about the future of their job spurred future-focused thinking and future-focused career behavior. This finding signals a Matthew effect of accumulated advantage, an effect that is often referred to as “the rich get richer and the poor get poorer” (Merton, 1968). In essence, the Matthew effect holds that people with a better starting position are more likely to succeed because of that starting position. Applied to the context of our study, the moderating effect of income adequacy can be interpreted as a Matthew effect in which people with sufficient means (i.e., a good starting position) are the ones who are able to look ahead and proactively build resources to master and change their career (i.e., succeed), while people with insufficient means may fall behind. While we did not find direct evidence for the latter, we do believe that a further exploration of the Matthew effect in job insecurity research is an important avenue for the future.

### Practical Implications

In addition to its theoretical contributions, our study has some clear implications for practice and policy. To date, most job insecurity interventions have been aimed at helping people cope with the stress and strain that arises from job insecurity –i.e., secondary and tertiary interventions (Hargrove et al., 2011). Our findings, however, suggest that engaging in proactive career behavior can decrease job insecurity itself, rather than its consequences. Therefore, job insecurity interventions should also make use of so-called primary interventions, in which workers can engage in proactive career behavior to prevent the onset or further development of job insecurity. Promising in this regard is research that shows that proactive behavior can be stimulated through interventions. For example, Glaub et al. (2014) showed that entrepreneurs’ proactive behavior could be changed through a personal initiative training, and Strauss and Parker (2015) showed that employees’ proactive skill development could be facilitated through training and development. These studies not only provide a positive outlook on the probability of success of enhancing proactive career behavior, but also provide excellent starting points for research and practice on how to set up a successful intervention.

In interventions aimed at increasing proactive career behaviors, a future focus should be central. For example, Strauss and Parker (2015) showed that their vision-focused proactivity intervention only led to higher proactive behavior among people with a strong future orientation. They speculated that the intervention made both the long-term benefits and the short-term costs of proactive behavior more salient, which may not stimulate people low in future orientation to become more proactive. Fortunately, we showed that the tendency to focus on the future is not a stable individual trait (e.g., Parker and Collins, 2010) but can also depend on people’s situation. In this specific case, having a sufficient income influenced participants’ focus on the future, but there are many other factors that may affect future focus, ranging from age to country of residence. For example, as cultures can differ in their long-term orientation, it may be that workers in future-oriented cultures have a greater tendency to engage in proactive career behavior than those in less future-oriented cultures (Hofstede, 2001; Probst et al., 2019). All in all, people’s future focus should be taken into account when designing and introducing interventions.

Importantly, our findings suggest that increasing job insecurity is not something that can only be achieved by individual actions. Flexicurity researchers have argued that also labor market policies that increase social security may reduce emotional distress about job loss (Berglund, 2015) and the moderating effect of income adequacy in the current study mimics this. At the same time, it remains to be seen whether providing income security can structurally compensate for job insecurity and its negative consequences, as there is little evidence for this assumption (cf. Berglund et al., 2014; Svetek, 2020). From a psychological perspective, this is not surprising: employment has more latent benefits than just income (e.g., social support, meaningfulness, identity, Jahoda, 1982) and providing income security may not be sufficient to fully compensate for the lack of job security. Nonetheless, if providing income adequacy can, indeed, help people to approach their career proactively in a sustainable way, it may be a worthwhile next step in creating alternative forms of security in an insecure labor market.

### Limitations

Although our study has several methodological strengths (e.g., a three-wave research design), the contributions of our study should be considered in light of a few limitations. First, the sample size is relatively small and we had to employ an estimate means procedure for the variables measured at Time 2 to ensure sufficient statistical power for testing the hypothesized model. While we believe our contributions to be meaningful, we also believe that the results should be interpreted with caution because of the relatively small sample size. To further strengthen the validity and generalizability of our results, it is of utmost importance that future research replicates our findings in a larger sample. Only then, solid conclusion can be drawn regarding the dynamic process between job insecurity and proactivity and the moderating effect of income adequacy. Second, our design is correlational and involved self-report measures, two factors that can possibly contribute to common method bias and limit the ability to draw causal conclusion. We attempted to minimize these threats by using a three-wave design in which we temporarily separated our predictor and process variables from the outcomes (see Podsakoff et al., 2003). Third, we should note that our results only apply to proactive career behavior and may thus not be generalized to all proactive coping behaviors. Moreover, the current study did not include measurement of alternative coping behaviors aimed at short-term solutions for workers, such as finding other (temporary) employment. Fourth, our sample consisted of self-employed professionals, meaning that generalizations to other workers have to be made with caution. Specifically, it is likely that self-employed professionals might differ in their proactivity and attitudes toward job insecurity compared to people in salaried employment.

### Conclusion

How do people think about and act upon the future of their work when this future is uncertain? This study provides initial evidence that even when the future of work is uncertain, focusing on that future remains important: it spurs proactive career behavior, which can lessen the expected likelihood of job loss. However, worrying about job loss can obstruct people’s cognitive functioning, and workers only seem to be able to translate their worries into a future focus and proactive actions when they are assured of an adequate income. Although more research is needed to substantiate these findings, our study showed that the multifaceted benefits that work offer also mean that various types of resources are needed to manage potential job loss. As such, policies that offer such resources may be instrumental to ensure optimal conditions for individual coping to succeed.

## Data Availability Statement

The data set, syntax, and output supporting the conclusions of this article are available in an online repository, accessible via https://osf.io/2e3cx/?view_only=a945fb5182394ed68950786008fc2cdc.

## Ethics Statement

The studies involving human participants were reviewed and approved by IRB University of Amsterdam; 2020-WOP-12462. The patients/participants provided their written informed consent to participate in this study.

## Author Contributions

MB organized the data collection. JK analyzed and interpreted the data. Both authors contributed to research conception and design, writing the manuscript, and approved the submitted version.

## Conflict of Interest

The authors declare that the research was conducted in the absence of any commercial or financial relationships that could be construed as a potential conflict of interest.

## Publisher’s Note

All claims expressed in this article are solely those of the authors and do not necessarily represent those of their affiliated organizations, or those of the publisher, the editors and the reviewers. Any product that may be evaluated in this article, or claim that may be made by its manufacturer, is not guaranteed or endorsed by the publisher.
